# Supplementary material for: Increasing Endoglin Deletion in Endothelial Cells Exacerbates the Severity of Brain Arteriovenous Malformation in Mouse
Source: Biomedicines. 2024 Jul 30;12(8):1691. doi: 10.3390/biomedicines12081691 (PMC11352040; doi:10.3390/biomedicines12081691)
Supplement: Supplementary file 1 [file biomedicines-12-01691-s001.zip › Figures and Tables.pdf]

Increasing Endoglin Deletion in Endothelial Cells Exacerbates the Severity of Brain Arteriovenous Malformation in Mouse

Zahra Shabani[1,2], Leandro Barbosa Do Prado[1,2], Rui Zhang[1,2], Wan Zhu[1,2], Sonali S Shaligram[1,2], Alka Yadav[1,2], Calvin Wang[1,2], Hua Su[1,2,*]

[1]Center for Cerebrovascular Research, University of California, San Francisco, San Francisco, CA, United States.
[2]Department of Anesthesia and Perioperative Care, University of California, San Francisco, San Francisco, CA, United States.

* Correspondence: Hua Su, Department of Anesthesia and Perioperative Care
University of California, San Francisco, 2540 23rd Street, Box 1363, San Francisco, CA 94143
Phone: 415-476-0141, Email: hua.su@ucsf.edu, ORCID: 0000-0003-1566-9877

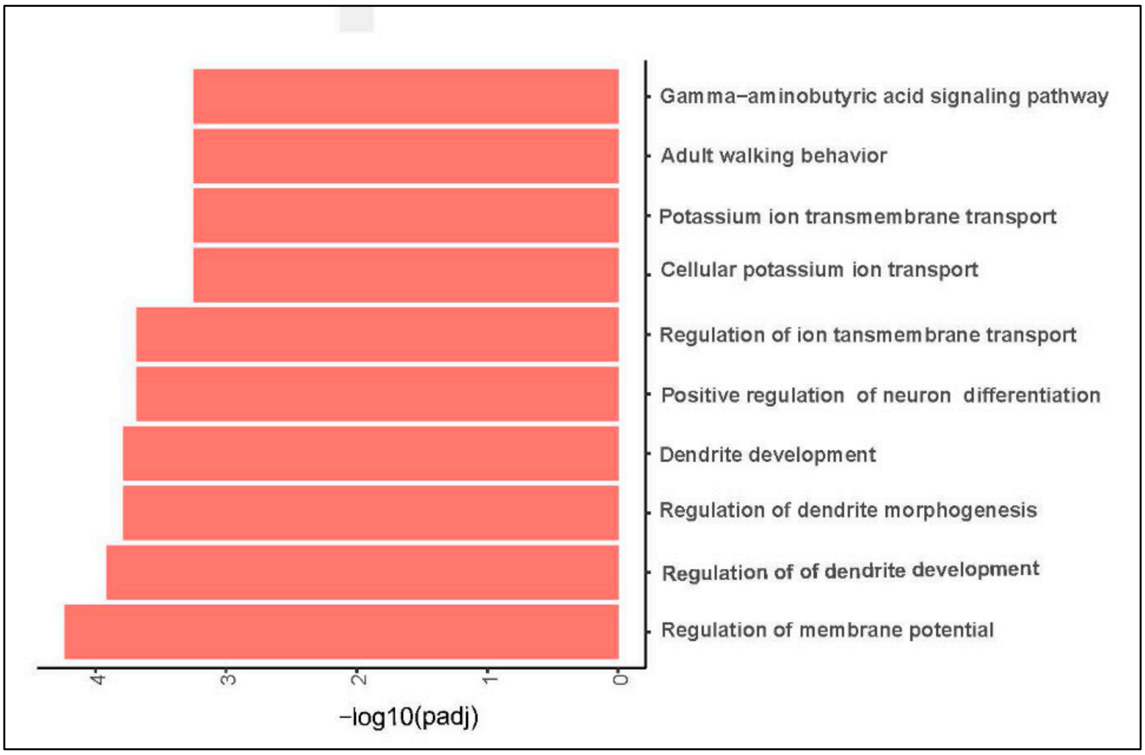

Supplementary Figure S1: Top 10 down-regulated biological pathways in icre mice compared to WT cre mice. None of these pathways is related to angiogenesis and inflammation.

Supplementary Table S1. Genes related to angiogenesis, innate immune response, and Leukocytes differentiation in top 100 upregulated Genes in bAVM of icre mice compared to WT cre mice.

Gene	Log2-fold change	Adjusted *p*-value
**Apobec3***	4.2	1.18E-78
**Gas5*****	3.6	9.47E-55
**Nr4a1*****	1.9	2.15E-12
**Cyr61*****	2.5	6.23E-12
**Klf2*****	1.7	2.11E-10
**Egr4*****	1.6	3.17E-07
**Egr2*****	2.8	2.98E-05
**Id1*****	1.2	0.0006
**Fcer1g****	1.1	0.0018
**Apold1*****	1.7	0.0039
**Egfl7*****	1	0.0061
**Klf4*****	1.4	0.0080
**Egr1*****	0.8	0.0165
**Stxbp2****	1.1	0.0195
**Gnas****	0.9	0.0202
**C1qa***	0.9	0.0389
**Nrarp*****	1.2	0.0389

*** Innate immune response**
**** Leukocytes differentiation**
***** Angiogenesis**
